# *PNPLA3* I148M Variant Impairs Liver X Receptor Signaling and Cholesterol Homeostasis in Human Hepatic Stellate Cells

**DOI:** 10.1002/hep4.1395

**Published:** 2019-07-15

**Authors:** Francesca Virginia Bruschi, Thierry Claudel, Matteo Tardelli, Patrick Starlinger, Fabio Marra, Michael Trauner

**Affiliations:** ^1^ Hans Popper Laboratory of Molecular Hepatology, Division of Gastroenterology and Hepatology, Department of Internal Medicine III Medical University of Vienna Vienna Austria; ^2^ Department of Surgery Medical University of Vienna Vienna Austria; ^3^ Clinical Pathophysiology Department University of Florence Florence Italy

## Abstract

The patatin‐like phospholipase domain‐containing protein 3 (*PNPLA3*) I148M variant predisposes to hepatic steatosis and progression to advanced liver injury with development of fibrosis, cirrhosis, and cancer. Hepatic stellate cells (HSCs) drive the wound healing response to chronic injury, and lack of liver X receptor (LXR) signaling exacerbates liver fibrogenesis by impairing HSC cholesterol homeostasis. However, the contribution of the I148M variant to this process is still unknown. We analyzed LXR expression and transcriptional activity in primary human HSCs and overexpressing LX‐2 cells according to *PNPLA3* genotype (wild type [WT] versus I148M). Here we demonstrate that LXRα protein increased whereas LXR target gene expression decreased during *in vitro* activation of primary human HSCs. Notably, LXRα levels and signaling were reduced in primary I148M HSCs compared to WT, as displayed by decreased expression of LXR target genes. Moreover, reduced expression of cholesterol efflux and enzymes generating oxysterols was associated with higher total and free cholesterol accumulation whereas endogenous cholesterol synthesis and uptake were diminished in I148M HSCs. Luciferase assays on LXR response element confirmed decreased LXR transcriptional activity in I148M HSCs; in contrast the synthetic LXR agonist T0901317 replenished LXR functionality, supported by adenosine triphosphate‐binding cassette subfamily A member 1 (ABCA1) induction, and reduced collagen1α1 and chemokine (C‐C motif) ligand 5 expression. Conversely, the peroxisome proliferator‐activated receptor gamma (PPARγ) agonist rosiglitazone had only partial effects on the LXR target gene ABCA1, and neither diminished expression of proinflammatory cytokines nor increased *de novo* lipogenic genes in I148M HSCs. *Conclusion:* As a consequence of reduced PPARγ activity, HSCs carrying I148M *PNPLA3* show impaired LXR signaling, leading to cholesterol accumulation. The use of a specific LXR agonist shows beneficial effects for diminishing sustained HSC activation and development of liver fibrogenesis.

**Abbreviations**
α‐SMA: α‐smooth muscle actin
ABCA1/G1: adenosine triphosphate‐binding cassette subfamily A/G, member 1
ACAT1: acyl‐coenzyme A:cholesterol acyltransferase 1
AP‐1: activator protein 1
CCL: chemokine (C‐C motif), ligand
CE: cholesterol ester
CH25H: cholesterol 25‐hydroxylase
CYP27A1: cytochrome P450 family 27, subfamily A, member 1 (sterol 27‐hydroxylase)
FASN: fatty acid synthase
FBS: fetal bovine serum
FC: free cholesterol
HMGCR: 3‐hydroxy‐3‐methylglutaryl coenzyme A reductase
HSC: hepatic stellate cell
IL‐8: interleukin‐8
KO: knockout
LDLR: low‐density lipoprotein receptor
LXR: liver X receptor
LXRE: liver X receptor response element
mRNA: messenger RNA
NAFLD: nonalcoholic fatty liver disease
NASH: nonalcoholic steatohepatitis
NPC1: Niemann‐Pick disease, type C intracellular cholesterol transporter 1
PNPLA3: patatin‐like phospholipase domain‐containing protein 3
PPARγ: peroxisome proliferator‐activated receptor gamma
PPRE: peroxisome proliferator response element
ROSI: rosiglitazone
RT‐PCR: real‐time polymerase chain reaction
SCD1: stearoyl‐coenzyme A desaturase 1
SREBP: sterol regulatory element‐binding protein
T09: T0901317
TC: total cholesterol
TGF: transforming growth factor
WT: wild type

Genetic factors are important for predisposition to and progression of metabolic liver diseases, such as nonalcoholic fatty liver disease (NAFLD).^1^, ^2^, ^3^ A single‐base polymorphism of the human patatin‐like phospholipase domain‐containing protein 3 (*PNPLA3*) gene (I148M/rs738409 C>G) encoding for adiponutrin is an independent risk factor for hepatic steatosis and susceptibility to liver disease progression, such as nonalcoholic steatohepatitis (NASH), alcoholic steatohepatitis, chronic hepatitis C, and hepatocellular carcinoma.^4^, ^5^ Hepatocellular injury, inflammation with recruitment of immune cells, and activation of hepatic stellate cells (HSCs) are key steps in the progression of NASH.^6^, ^7^, ^8^ HSC activation includes a metabolic and phenotypic switch from quiescent fat‐storing cells toward highly proliferative and reactive myofibroblast‐like HSCs, the key drivers of the fibrotic wound healing response.^9^ We have recently uncovered a fundamental role of *PNPLA3* in HSCs to achieve a fully activated phenotype and the impact of the I148M genetic variant to promote inflammatory cytokine release, with subsequent immune cell recruitment, proliferation, and expression of profibrogenic genes in human HSCs.^10^

Impaired cholesterol homeostasis leads to free cholesterol (FC) accumulation in HSCs, thus predisposing to toll‐like receptor 4 (TLR4) signaling, down‐regulation of bone morphogenetic protein and activin membrane‐bound inhibitor (BAMBI), and transforming growth factor (TGF)‐β‐mediated fibrogenesis.^11^, ^12^, ^13^ Two members of the nuclear receptor superfamily, the liver X receptors (LXRs) α and β nuclear receptor subfamily 1, group H, member 3 (NR1H3)/LXRα^14^ and NR1H2/LXRβ,^15^ have a pivotal role in control of cholesterol homeostasis and lipid metabolism throughout the human body.^16^ The transcriptional activity of these nuclear receptors is regulated by metabolites of cholesterol, known as oxysterols, and by retinoid X receptor availability to form heterodimer complexes.^17^ Remarkably, the two LXR isotypes display distinct function and tissue distribution throughout the body; β is ubiquitously expressed, whereas α is restricted to metabolic organs.^18^, ^19^ In the liver, transcripts of LXRα and LXRβ were found in both parenchymal and nonparenchymal cells.^20^, ^21^, ^22^ LXRs have received considerable attention as possible candidates for therapeutic approaches in metabolic diseases due to LXRα‐mediated induction of bile acid synthesis, hepatic *de novo* lipogenesis, and reduction of high‐density lipoprotein cholesterol in LXRα knockout (KO) mice.^23^, ^24^, ^25^, ^26^, ^27^ In particular, lack of LXR exacerbated liver fibrosis in two different mouse models of chronic liver injury. HSCs isolated from LXR KO mice showed altered lipid partitioning, as reported, by enlarged lipid droplet accumulation compared to wild‐type (WT) cells and enhanced expression of inflammatory and fibrotic molecules, such as collagen1α1, monocyte chemoattractant protein 1, and platelet‐derived growth factor β.^21^ Of note, PNPLA3 expression is controlled by LXR/sterol regulatory element‐binding protein 1c (SREBP‐1c), thus suggesting the tight regulation of this protein along with *de novo* lipogenesis, at least in hepatocytes.^28^ Moreover, peroxisome proliferator‐activated receptor gamma (PPARγ) positively controls LXRα, thus favoring cholesterol efflux from foam cells.^29^ HSCs expressing the variant of *PNPLA3* (I148M) showed that decreased PPARγ signaling up‐regulates proinflammatory molecule production and release through enhanced transcriptional activity of activator protein 1 (AP‐1) by means of c‐Jun N‐terminal kinase.^10^ These observations encouraged us to explore the hypothesis that I148M *PNPLA3* might impair the crosstalk between PPARγ and LXR, a master regulator of *de novo* lipogenesis and cholesterol homeostasis in the liver.^26^, ^27^

Therefore, our aim was to evaluate whether the genetic variant of *PNPLA3* contributes to altered LXR signaling in human HSCs in order to further uncover potential novel therapeutic strategies targeting the molecular and metabolic changes in patients with *PNPLA3* I148M.

## Materials and Methods

### Human HSC Isolation and Cell Culture

HSCs were isolated from donor livers unsuitable for transplantation or liver resections for metastasis of colorectal cancer, as approved by the ethics committees of the University of Florence^10^ and the Medical University of Vienna (Ethic Committee number 2032/2013). HSCs were seeded on uncoated plastic dishes and cultivated with Iscove’s modified Dulbecco’s medium (EuroClone, Italy) supplemented with 20% fetal bovine serum (FBS), 1% glutamine 200 mM, sodium pyruvate 100 mM, nonessential amino acid solution 100X, and antibiotic antimycotic solution 100X (Gibco Life Technologies, Carlsbad, CA). Primary HSCs between passages one and eight were used for this study. For comparison, primary WT and I148M *PNPLA3* HSCs were used at the same passage. The genotype of each isolated HSC line has been analyzed by real‐time polymerase chain reaction (RT‐PCR) for the I148M single‐nucleotide polymorphism, as done routinely in our clinical research center. Only homozygote genotypes (n = 3) were used in this study (C/C as WT and G/G as I148M).

Stably overexpressing WT and I148M LX‐2 were generated in our laboratory, as described,^10^ and cultured with Dulbecco’s modified Eagle’s medium (Gibco) supplemented with 5% FBS and 1% penicillin/streptomycin solution (EuroClone).

For cell culture treatments, cells deprived of FBS for 24 hours were stimulated with 10 μM of specific LXR agonist (T0901317 [T09]; Sigma‐Aldrich), inverse agonist (SR9238; Tocris), antagonist (GSK2033; Tocris), and PPARγ agonist (rosiglitazone [ROSI]; Sigma‐Aldrich) for an additional 24 hours.

### Western Blot Analysis

Whole‐cell extracts were obtained using radio immunoprecipitation assay buffer (150 mM NaCl, 1% Triton X‐100, 0.5% sodium deoxycholate, 0.1% sodium dodecyl sulfate, and 50 mM Tris, pH 8.0) containing complete ethylene diamine tetraacetic acid‐free protease inhibitor cocktail tablets (Roche Diagnostics GmbH, Germany) and phosphatase inhibitors (20 mM b‐glycerophosphate, 10 mM 4‐nitrophenylphosphate, and 50 mM sodium vanadate; Sigma‐Aldrich). Primary antibodies as follows were diluted in 3% bovine serum albumin trishydroxymethylaminomethane‐buffered saline Tween 1X solution at different concentrations: rabbit polyclonal to SREBP‐2, 3‐hydroxy‐3‐methylglutaryl coenzyme A reductase (HMGCR), and LXRα (Novus NB300‐612) (1:500; all from Santa Cruz Biotechnology); mouse monoclonal to LXRβ (1:500; Santa Cruz Biotechnology); rabbit monoclonal to low‐density lipoprotein receptor (LDLR) (1:1,000; Abcam, Cambridge, United Kingdom); and rabbit polyclonal to calnexin and β‐actin (1:10,000; Sigma‐Aldrich). Proteins were detected by enhanced chemiluminescence (GE Amersham, Arlington Heights, IL).

### Cholesterol Measurements

For lipid extraction and analysis, approximately 1 × 10^6^ cells carrying the two distinct *PNPLA3* genotypes were harvested and resuspended in 200 μL solution containing chloroform:isopropanol:nonyl phenoxypolyethoxylethanol (NP‐40) (7:11:0.1) in a microhomogenizer. Thereafter, the extract was centrifuged for 10 minutes at 15,000*g* and the liquid phase was isolated and dried at 50°C for 30 minutes to remove traces of organic solvent. Once dried, the lipids were dissolved in the specific assay buffer, and total cholesterol (TC) and FC were quantified using a colorimetric assay kit (ab65359; Abcam) according to the manufacturer’s instructions.

### Luciferase Assay

Stably overexpressing WT or I148M LX‐2 were seeded in a 24‐well plate and transiently transfected with 0.3 μg/well of an AP‐1, LXR response element (LXRE)‐luciferase or peroxisome proliferator response element (PPRE)‐luciferase construct using Fugene transfection reagent (Promega, Madison, WI) in sterile Opti‐modifed Eagle’s medium for 12 hours. Complete medium was then replaced for an additional 24 hours, and the cells were lysed using a solution of 4% Triton X‐100, glycylglycine 100 mM, MgSO_4_ 100 mM, and ethylene glycol tetraacetic acid 250 mM for 1 hour at room temperature on a shaker platform. The extracts were then combined with the solution containing the substrate (luciferin 2.5 mM and adenosine triphosphate 20 mM; Sigma‐Aldrich) and analyzed with a luminometer (Lumat LB950; EG&G Berthold, Germany). AP‐1‐luciferase was bought from Takara Bio USA; pcDNA3.1‐PPARγ was a gift from Dr. V.K. Chatterjee (Department of Medicine, University of Cambridge, Addenbrooke’s Hospital, Cambridge, United Kingdom); and pSG5‐LXRα, pSG5‐LXRβ, and DR4‐thymidine kinase luciferase were generous gifts from Dr. David Mangelsdorf (University of Texas Southwestern Medical Center, Dallas, TX). The pSG5 Vector is a eukaryotic expression vector constructed by combining pKCR2 and the Stratagene pBS vector. In this case either human LXRalpha or Beta were cloned into this vector.

### Statistical Analysis

Unless otherwise indicated, data presented as bar graphs are the mean ± SD of at least two independent experiments performed in triplicates. Three separated primary HSC isolations and three different clones carrying either WT or I148M *PNPLA3* genotypes were used (n = 3 for each genotype). Statistical analysis was performed using GraphPad Prism (La Jolla, CA). The unpaired Student *t* test was used when two groups were compared, and two‐way analysis of variance was used for multiple groups. *P* < 0.05 was considered statistically significant.

## Results

### Primary Human HSCs Carrying the *PNPLA3* I148M Variant Display Reduced LXRα Expression and Transcriptional Activity Compared to WTs

**Figure 1:**
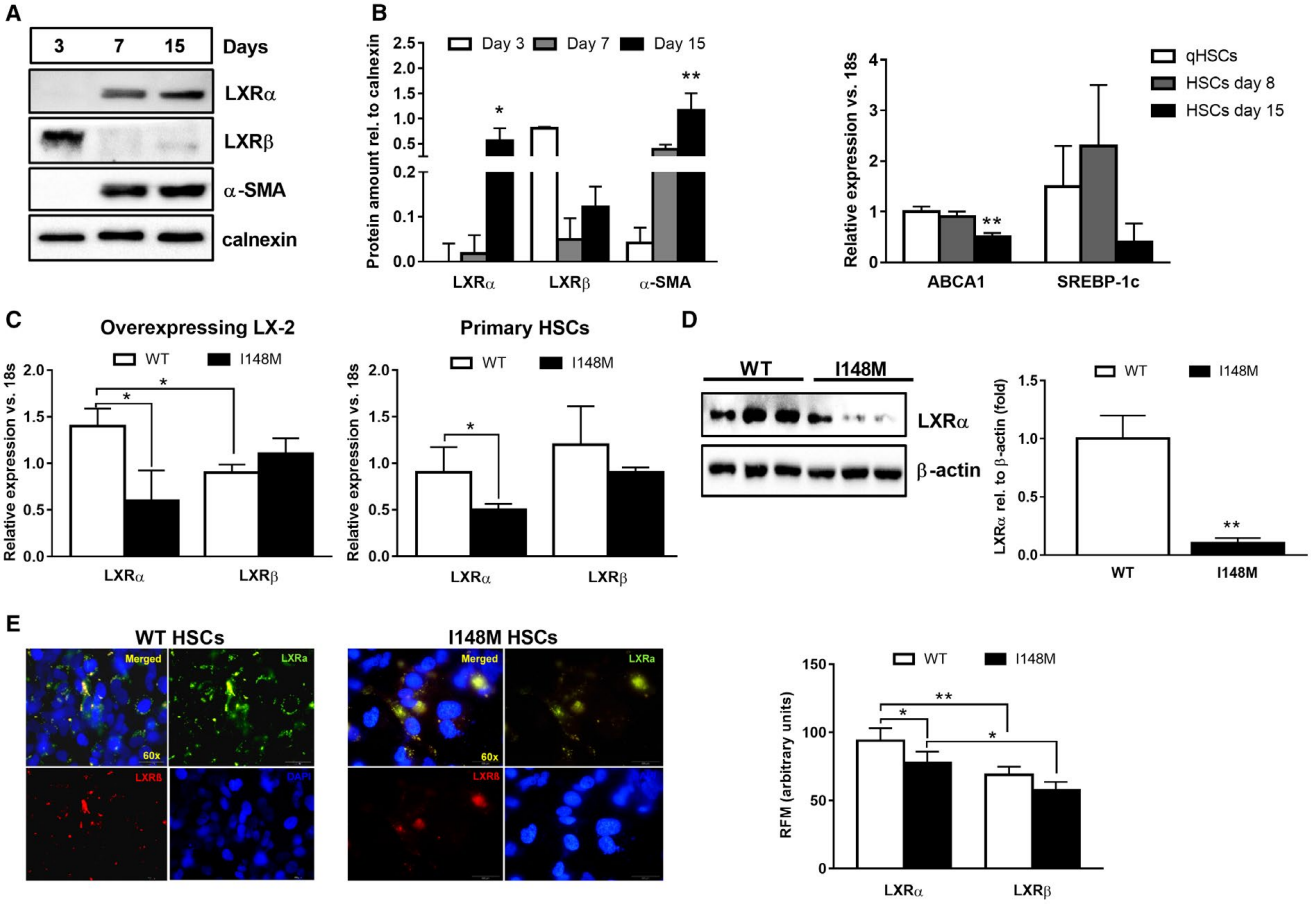
Decreased LXRα transcriptional activity during human HSC activation and in *PNPLA3* I148M HSCs compared to WT. Human primary HSCs isolated from liver resections and cultivated *in vitro*, as described in Materials and Methods. All bar graphs show mean values ± SD. (A) During *in vitro* activation (3, 7, and 15 days after cell isolation), cells were harvested and total protein extracts were used to detect LXRα, LXRβ, α‐SMA, and calnexin by western blotting. Blots are representative of three independent cell isolations. Densitometry analysis of blots was performed using ImageJ software, and results were normalized to calnexin. Data presented as protein amount relative to calnexin. Open bars show results for 3 days, gray bars for 7 days, and black bars for 15 days after isolation. *P < 0.05, **P < 0.01 versus day 3. (B) ABCA1 and SREBP‐1c mRNA expression analyzed by RT‐PCR and data normalized to 18s. Freshly isolated cell data are depicted in white bars, 8 days after isolation data in gray bars, and 15 days after isolation in black bars (n = 3 independent isolations). **P < 0.01 versus qHSCs. (C,D) Whole‐cell extracts isolated from primary fully activated HSCs and stably overexpressing LX‐2 (n = 3 for each *PNPLA3* genotype), as indicated in the figure. Expression of both LXRα and LXRβ analyzed by RT‐PCR and western blotting. Data normalized to 18s and β‐actin for mRNA and protein, respectively. Open bars refer to *PNPLA3* WT HSCs and closed bars to I148M HSCs. Representative blots displayed. *P < 0.05, **P < 0.01 versus WT HSCs. (E) Immunofluorescence staining shows intracellular localization of LXRα (Alexa 488, green) and LXRβ (Alexa 568, red) in untreated human primary HSCs (n = 3 for each *PNPLA3* genotype). DAPI (blue) stains the nuclei. Merged (yellow) picture shows overlapping green and red channels. Magnification ×60, and only representative images displayed. Quantitative RFM for LXRα (green channel) and LXRβ (red channel) was calculated from representative pictures (n = 6 from each glass coverslip) using ImageJ software. Open bars refer to *PNPLA3* WT HSCs and closed bars to I148M HSCs. *P < 0.05 and **P < 0.01, as indicated in the bar graph. Abbreviations: DAPI, 4′,6‐diamidino‐2‐phenylindole; qHSC, freshly isolated HSC; rel., relative; RFM, relative fluorescence mean.

Because the nuclear receptor LXR exerts anti‐inflammatory and antifibrotic actions in HSCs and the lack of LXR results in exacerbated liver fibrosis *in vivo*,^21^ we investigated the expression pattern of the two known isotypes LXRα and LXRβ during human primary HSC activation *in vitro*. Interestingly, the LXRα protein amount increased progressively along with the profibrogenic marker α‐smooth muscle actin (α‐SMA) (Fig. 1A) during HSC activation, whereas LXRβ was more abundant in the early phases (day 3 after isolation). In order to evaluate whether this finding corresponded to increased transcriptional LXR activity, we measured two key LXR target genes, adenosine triphosphate‐binding cassette subfamily A, member 1 (ABCA1) and SREBP‐1c. Interestingly, both LXR target genes were down‐regulated, as reflected by significantly reduced ABCA1 (*P* < 0.001) expression in myofibroblast‐like cells (HSCs 15 days after isolation) compared to quiescent HSCs (or freshly isolated HSCs; Fig. 1B), thus indicating reduced LXR functionality during HSC activation.

Given the influence of the *PNPLA3* genetic variant to the altered phenotype of HSC,^10^ we next compared the expression of LXRα and LXRβ, evaluated by RT‐PCR, western blotting, and immunofluorescence (IF) intracellular staining, among primary HSCs with the two different *PNPLA3* genotypes. Interestingly, we found significantly lower LXRα expression in *PNPLA3* I148M cells compared to WT HSCs (Fig. 1C‐E).

In a pilot series, we also evaluated the amount of LXRα IF staining in NASH liver biopsies with different degrees of fibrosis and classified according to WT or I148M *PNPLA3* genotype. Total LXRα expression was higher in patients carrying the variant compared to WT and correlated with a higher degree of steatosis but only partially colocalized with the HSC activation marker α‐SMA (data not shown). Collectively, our data established that LXR transcriptional activity is lowered during human HSC activation and diminishes in the presence of the *PNPLA3* I148M variant compared to WT HSCs.

### Decreased LXR Signaling Disrupts Cholesterol Homeostasis in Human HSCs With *PNPLA3* I148M

**Figure 2:**
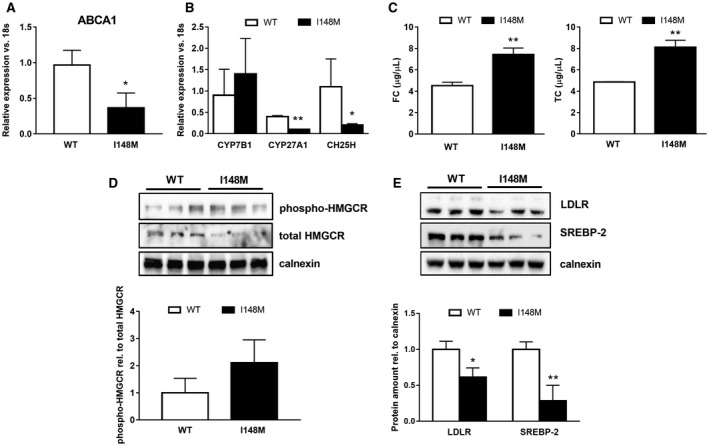
Decreased LXR signaling disrupts cholesterol homeostasis in *PNPLA3* I148M carrying human HSCs. Human primary HSCs isolated from liver resections and cultivated *in vitro* and LX‐2 stably overexpressing cells (n = 3 for each *PNPLA3* genotype) obtained, as described in Materials and Methods. All bar graphs show mean values ± SD. (A,B) Expression of cholesterol efflux transporter ABCA1 and cholesterol‐metabolizing enzymes CYP7B1, CYP27A1, and CH25H analyzed by RT‐PCR and normalized to 18s in untreated primary HSCs carrying either the WT or the variant of *PNPLA3* (n = 3 for each genotype); *P < 0.05, **P < 0.01. (C) TC and FC amounts quantified by using colorimetric quantitative assay on total lipid extracts collected from HSCs carrying either WT or I148M *PNPLA3*. Data shown are representative of two independent lipid extractions normalized to internal standards (Abcam); **P < 0.01. (D,E) Total protein extracts were isolated from primary HSCs carrying either WT or I148M *PNPLA3* (n = 3 for each genotype) and analyzed by western blotting for SREBP‐2, LDLR, phospho‐HMGCR, and total HMGCR. Densitometry analysis was performed using ImageJ software, and data were normalized to calnexin. Data presented as protein amount relative to calnexin. Phospho‐HMGCR was normalized to total HMGCR and expressed as protein ratio. Open bars refer to PNPLA3 WT HSCs and closed bars to I148M HSCs (n = 3 for each genotype); *P < 0.05, **P < 0.01 versus WT carriers. Abbreviations: phospho‐, phosphorylated; rel., relative.

To explore the functional impact of reduced LXR amount in *PNPLA3* I148M HSCs, we analyzed downstream targets of LXR, which could be separated into the following two main categories^27^: (1) cholesterol efflux transporters ABCA1 and ABCG1^30^, ^31^ and (2) *de novo* lipogenic genes (SREBP‐1c, fatty acid synthase [FASN], and stearoyl‐coenzyme A desaturase 1 [SCD1]). Gene expression analysis showed a significantly lower ABCA1 expression in I148M HSCs (Fig. 2A), with no significant effect on ABCG1 and ABCG4 (Supporting Fig. S1). In addition, because oxysterols are natural ligands of LXR, we measured the gene expression of three pivotal enzymes producing LXR‐activating oxysterols. Interestingly, primary HSCs carrying I148M *PNPLA3* showed a significant reduction of cytochrome P450 family 27, subfamily A, member 1 (CYP27A1; sterol 27‐hydroxylase) and cholesterol 25‐hydroxylase (CH25H) expression (Fig. 2B), suggesting a reduction in oxysterol generation.

Intracellular FC accumulation sensitizes HSCs to profibrogenic actions of TGF‐β stimulation and promotes liver fibrosis development *in vivo*.^11^ To determine whether the genetic variant of *PNPLA3* has consequences on cholesterol metabolism and homeostasis in HSCs, we measured the TC and FC content in stably overexpressing WT and I148M LX‐2. First, we found a significant increase of TC (*P* < 0.01) and FC (*P* < 0.01) content in cells carrying the PNPLA3 variant (Fig. 2C). Endogenous cholesterol synthesis is regulated by HMGCR, which represents the rate‐limiting step of the mevalonate pathway, and due to its central role, HMGCR is finely tuned and turned off by phosphorylation on a specific site (S872). Consistently, the ratio of phosphorylated HMGCR/total HMGCR is higher in HSCs carrying the variant (Fig. 2D), and this is supported by decreased messenger RNA (mRNA) as well (Supporting Fig. S2). Thereafter, we investigated the transcriptional activity of SREBP‐2, a key sensor and modulator of intracellular cholesterol content. SREBP‐2 gene expression (Supporting Fig. S1) and protein levels (Fig. 2E) were significantly lowered (*P* < 0.05, *P* < 0.01, respectively) in HSCs carrying the *PNPLA3* variant, in line with higher TC and FC content. Accordingly, SREBP‐2 downstream target LDLR was significantly diminished at protein levels (Fig. 2E). Moreover, expression of *de novo* lipogenic genes and downstream targets of LXR, such as FASN, SCD1, and SREBP‐1c, decreased in primary as well as in overexpressing PNPLA3 variant HSCs (Supporting Fig. S2).

Notably, expression of acyl‐coenzyme A:cholesterol acyltransferase 1 (ACAT1) and Niemann‐Pick disease, type C intracellular cholesterol transporter 1 (NPC1) was profoundly down‐regulated (*P* < 0.01) in primary HSCs carrying the *PNPLA3* variant, thus supporting that augmented FC content might result from decreased esterification to cholesterol esters (CEs) (Supporting Fig. S1). Therefore, based on the latter observations, we might conclude that LXR signaling is decreased due to diminished endogenous ligand generation, leading to accumulation of both TC and FC in HSCs expressing the genetic variant of *PNPLA3*.

### LXR Transcriptional Activity is Impaired in *PNPLA3* I148M HSCs due to LACK of Upstream PPARγ Functionality

**Figure 3:**
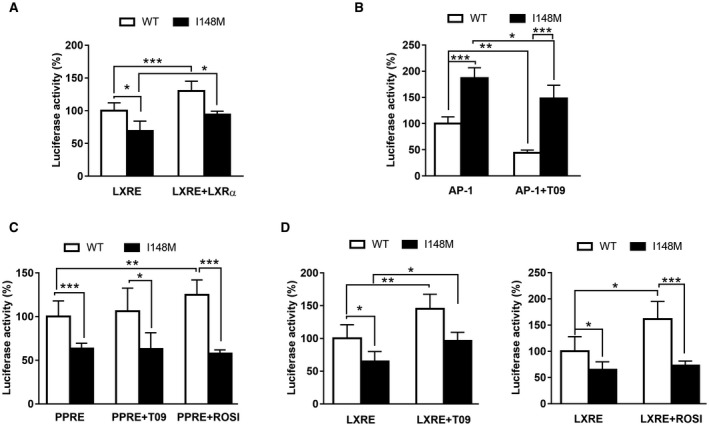
LXR transcriptional activity is lower due to impaired PPARγ1 functionality in HSCs carrying *PNPLA3* I148M. LX‐2 cells stably overexpressing WT or I148M *PNPLA3* transiently transfected with either LXRE, AP‐1, or PPRE plasmids to measure LXR, AP‐1, or PPARγ transcriptional activity, respectively. After 48 hours, luciferase activity (expressed as percentage) was measured using a luminometer and normalized to total protein content, as described in Materials and Methods. All bar graphs show mean values ± SD. (A) LXRE‐luciferase activity was measured either in untreated HSCs or in the presence of nuclear receptor LXR (LXRE+LXR) for 24 hours. (B) AP‐1‐luciferase activity was measured either in untreated HSCs or in the presence of the synthetic LXR agonist T09 for 24 hours. (C) PPARγ‐luciferase activity was measured in untreated (PPRE) or previously treated HSCs with either T09 (PPRE+T09) or rosiglitazone (PPRE+ROSI) for 24 hours. (D) LXRE‐luciferase activity was measured in untreated (LXRE) or in pretreated HSCs with either 10 nM of T09 (LXRE+T09) or rosiglitazone (LXRE+ROSI) for 24 hours. All bar graphs are representative of four independent transfections performed in triplicates. *P < 0.05, **P < 0.01, and ***P < 0.001, as indicated in the bar graphs. Open bars refer to *PNPLA3* WT HSCs and closed bars to I148M HSCs (n = 3 for each genotype).

Ozasa et al.^24^ and Chinetti and coworkers^30^ reported the tight connection between PPARγ and LXR axes in controlling ABCA1 expression in human macrophages. Along with the findings published from our group on impaired PPARγ signaling in the *PNPLA3* variant promoting higher proinflammatory cytokine release,^10^ we further explored whether PPARγ1 (the major isoform in HSCs) inhibition was contributing also to the reduced LXR transcriptional activity in cells carrying the *PNPLA3* genetic variant. Notably, in comparison to WT cells, LXRE‐luciferase reporter activity was reduced and could not be significantly induced even in the presence of exogenous LXR in I148M HSCs (Fig. 3A). In addition, AP‐1‐luciferase activity increased significantly (2‐fold) in cells expressing I148M *PNPLA3* and was inhibited after LXR agonist T09 treatment (Fig. 3B). Furthermore, analysis of the PPRE using the luciferase assay revealed that HSCs carrying the *PNPLA3* variant showed almost a 50% reduction for PPARγ transcriptional activity, which could not be rescued by using either LXR or PPARγ synthetic agonists T09 and ROSI, respectively (Fig. 3C). In contrast, endogenous LXR stimulation by T09 treatment induced LXRE‐luciferase activity in *PNPLA3* WT and I148M cells and virtually normalized LXR signaling in both *PNPLA3* genotypes (Fig. 3D, panel LXRE vs. LXRE+T09; no significant difference between WT and I148M after T09), whereas ROSI increased LXRE‐luciferase activity only in WT HSCs (Fig. 3D, LXRE+ROSI; significant difference between WT and I148M in LXRE‐luciferase activity). Taken together, these data suggest that the lack of endogenous LXR ligand could explain impaired LXR signaling in I148M *PNPLA3* cells and that PPARγ ligand increases LXR signaling only in WT cells.

### Synthetic LXR Agonist T09 Induces LXR Downstream Targets Independently From PPARγ in Both WT and I148M *PNPLA3* HSCs

**Figure 4:**
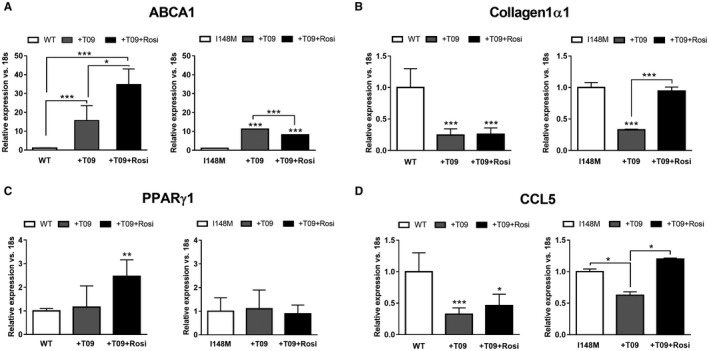
LXR agonist T09 alone restores ABCA1 and attenuates inflammatory and fibrogenic markers independently from PPARγ in HSCs with the *PNPLA3* I148M variant. LX‐2 stable overexpressing WT or I148M *PNPLA3* untreated or treated for 24 hours with either T09 (+T09) or the combination T09 and ROSI (+T09+Rosi). mRNA expression of (A) ABCA1, (B) collagen1α1, (C) PPARγ1 and (D) CCL5 and analyzed by RT‐PCR and normalized to 18s. White bars show untreated HSCs, gray bars show HSCs treated with T09, and black bars show HSCs treated with T09 or the combination (+T09+Rosi). All bar graphs show mean values ± SD. *P < 0.05, **P < 0.01, and ***P < 0.001 versus untreated WT or I148M HSCs (n = 3 for each genotype).

In order to further explore and confirm our observations on the different responses induced by T09 and ROSI on LXRE, we stimulated cells with T09 and compared LXR target gene expression in WT and I148M *PNPLA3* HSCs. Stimulation with the synthetic LXR agonist T09 alone showed a significant induction of ABCA1 and reduction of profibrogenic markers collagen1α1 and chemokine (C‐C motif) ligand 5 (CCL5) in WT‐expressing HSCs (*P* < 0.001; Fig. 4, left panels, gray bars). Similarly, T09 also significantly promoted ABCA1 expression and decreased collagen1α1 and CCL5 in I148M variant HSCs (*P* < 0.001; Fig. 4, right panels, gray bars). Interestingly, the addition of ROSI to T09 showed further induction of ABCA1 expression only in WT cells (*P* < 0.001; Fig. 4, left panels, black bars) and displayed consistent reduction of collagen1α1 and CCL5 as the stimulation with T09 alone, supporting the hypothesis that PPARγ positively affects LXR target gene expression. In contrast to WT *PNPLA3* HSCs, *PNPLA3* I148M variant cells stimulated with T09 in combination with ROSI did not display higher ABCA1 and diminished collagen1α1 and CCL5 (*P* < 0.05 and *P* < 0.001, respectively; Fig. 4, right panels, black bars). These observations, therefore, suggest that T09 induces LXR signaling in HSCs carrying I148M *PNPLA3* whereas ROSI has these beneficial effects only in WT *PNPLA3* HSCs.

### Specific LXR Inverse Agonist SR9238 and Antagonist GSK2033 Block LXR Signaling in WT and I148M *PNPLA3* HSCs

**Figure 5:**
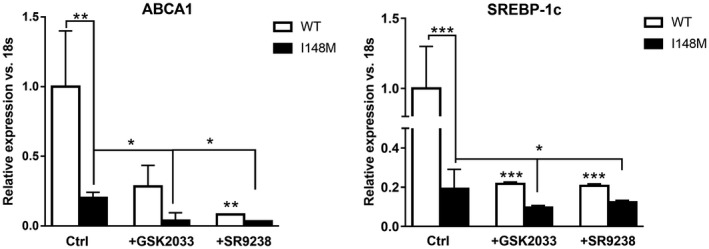
LXR inverse agonist SR9238 and antagonist GSK2033 blocked T09‐induced LXR signaling in human HSCs. Whole mRNA extract collected from untreated (ctrl) stably overexpressing LX‐2 carrying either WT or I148M *PNPLA3*, treated with LXR inverse agonist (SR9238) or LXR antagonist (GSK2033) for 24 hours. Expression of ABCA1 and SREBP‐1c analyzed by RT‐PCR and normalized to 18s. I148M expression data were normalized to WT ctrl. All bar graphs show mean values ± SD. *P < 0.05, **P < 0.01, and ***P < 0.001 versus untreated WT HSCs or as indicated in the graph. Abbreviation: Ctrl, control.

Because T09 is also known to have LXR off‐targeting effects involving farnesoid X receptor (FXR) and pregnane X receptor (PXR)^32^, ^33^ and to evaluate the specificity of LXR signaling and the positive effects shown by the LXR agonist T09 in human HSCs, we used a specific synthetic LXR inverse agonist (SR9238) and LXR antagonist (GSK2033) to block LXR signaling in both WT and I148M HSCs. In WT HSCs, both SR9238 and GSK2033 significantly reduced ABCA1 and SREBP‐1c expression (*P* < 0.05, *P* < 0.01, respectively; Fig. 5, white bars). In contrast to the positive actions of T09, both GSK2033 and SR9238 resulted in a significant decrease in ABCA1 and SREBP‐1c expression also in I148M *PNPLA3* HSCs (*P* < 0.05, *P* < 0.01, respectively; Fig. 5, black bars). Taken together, these data established the role of LXR signaling in the regulation mediated by the T09 agonist.

### ROSI Promotes LXR Signaling and Decreases Inflammatory Cytokine Expression Only in HSCs Expressing WT *PNPLA3*

**Figure 6:**
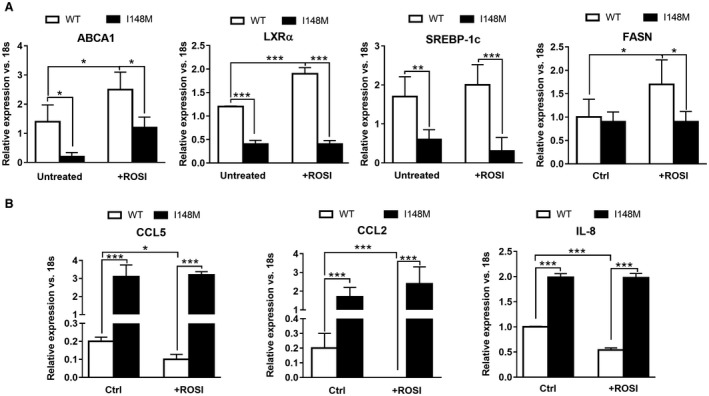
ROSI up‐regulates LXRα prolipogenic genes and reduces inflammatory cytokine expressions only in WT but not I148M HSCs. Whole mRNA extract collected from untreated stably overexpressing LX‐2 carrying either WT or I148M *PNPLA3* or treated with ROSI for 24 hours. All bar graphs show mean values ± SD. (A) Expression of ABCA1, LXRα, SREBP‐1c, and FASN analyzed by RT‐PCR and normalized to 18s. (B) Expression of CCL5, CCL2, and IL‐8 analyzed by RT‐PCR and normalized to 18s. Data displayed represent two independent experiments performed in triplicates. White bars refer to *PNPLA3* WT HSCs and black bars to I148M (n = 3 for each genotype); *P < 0.05, **P < 0.01, and ***P < 0.001. Abbreviation: Ctrl, control.

**Figure 7:**
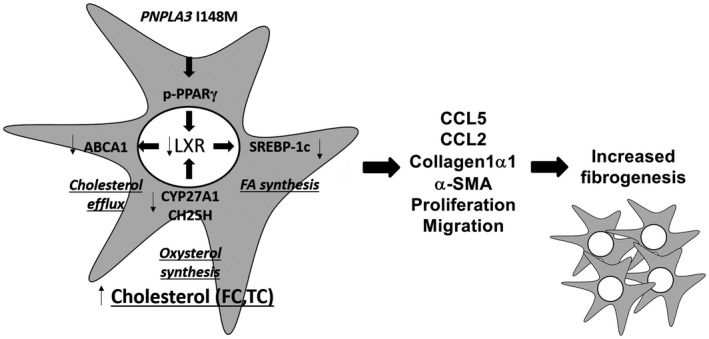
Summary representation of impaired LXRα signaling resulting in altered TC and FC accumulation in human HSCs carrying I148M *PNPLA3*. Activated human HSCs carrying I148M have decreased PPARγ transcriptional activity due to phosphorylation on specific inhibitory residue (p‐PPARγ), which directly impairs LXR target gene expression involved in cholesterol and lipid metabolism (ABCA1, SREBP‐1c, FASN). As such, reduced cholesterol efflux through ABCA1 leads to TC and FC accumulation, accompanied by reduced endogenous cholesterol synthesis (SREBP‐2, HMGCR) and decreased extracellular uptake (LDLR). Reduced PPAR/LXR signaling axe decreases the anti‐inflammatory ability of these nuclear receptors, thus resulting in higher expression of profibrogenic features (CCL5, CCL2, collagen1α1, α‐SMA, increased migration and proliferation).

Data collected by our group have highlighted the impact of the genetic variant of *PNPLA3* in inducing a proinflammatory phenotype in human HSCs.^10^ Because activation of LXRs attenuates inflammation and inflammatory gene expression in many cell types^34^ and ROSI positively regulates LXRE in WT but not in I148M‐expressing cells (Figs. 3D and 4), we speculated that activation of PPARγ controls LXRs and the downstream targets in WT *PNPLA3* cells but not in I148M cells. To test the hypothesis, we next treated HSCs with ROSI only (Fig. 6). Interestingly, PPARγ synthetic agonist up‐regulated ABCA1 expression in both WT and I148M HSCs but LXRα only in WT cells (Fig. 6A), thus suggesting that the partial recovery of LXRE shown in Fig. 3D resulted in induction of specific gene sets. Consistently, ROSI positively influenced the expression of the LXRα downstream lipogenic gene FASN only in WT *PNPLA3*‐expressing HSCs, whereas SREBP‐1c in I148M cells was not increased (Fig. 6A), thus supporting that PPARγ/LXR signaling crosstalk was blunted in I148M cells, which might be partially recovered by ROSI. Importantly, the treatment significantly decreased (*P* < 0.001) expression of proinflammatory cytokines, such as CCL2, CCL5, and interleukin‐8 (IL‐8), only in WT but not I148M HSCs (Fig. 6B), consistent with our observations on the posttranslational modification of PPARγ by phosphorylation in HSCs carrying I148M^10^ and the luciferase data (Fig. 3D). Collectively, our observations suggest that I148M *PNPLA3* lacks LXR activation, probably due to weak oxysterol synthesis, resulting in accumulation of FC and TC. In addition, stimulation with synthetic agonists showed distinct effects on PPARγ and LXR target gene expressions, suggesting that lack of PPARγ activation upstream represses LXR activity whereas direct activation of LXR with its specific agonist T09 results in significantly higher ABCA1 and less collagen1α1 and CCL5 expression in HSCs carrying the I148M variant (Fig. 7).

## Discussion

Our findings report novel mechanistic aspects explaining the impact of the genetic variant I148M on LXR nuclear receptor signaling and cholesterol metabolism in human HSCs. The genetic variant of *PNPLA3* results in phosphorylation of PPARγ on specific inhibitory residue, down‐regulating its transcriptional activity, and impairing the downstream functionality of the two isotypes of LXRs. In addition, decreased LXR signaling diminishes intracellular cholesterol efflux (through ABCA1) and *de novo* lipogenesis (through SREBP‐1c) and promotes accumulation of toxic FC and TC, which are not metabolized to oxysterols (by CYP27A1, CYP7B1 [known also as 25‐hydroxycholesterol 7‐alpha‐hydroxylase], and CH25H) or esterified to CEs (by ACAT1). Altogether, HSCs carrying I148M *PNPLA3* show enhanced expression of proinflammatory mediators (CCL2, CCL5, and IL‐8), proliferation, and migration capability.^10^

Oxysterols represent the natural ligands for LXR nuclear receptors^25^ and have been shown to control lipid metabolism and cholesterol homeostasis in different cell types and tissues.^20^ In particular, LXRs regulate lipogenic and inflammatory pathways in HSCs because lack of LXR signaling resulted in impaired lipid partitioning and increased fibrogenic and inflammatory cytokine expression *in vitro* and *in vivo*.^21^ Analyzing LXRs during early *in vitro* activation of freshly isolated human HSCs revealed that LXRα increased along with α‐SMA whereas the β isotype decreased (Fig. 1A). Nevertheless, LXR downstream targets are down‐regulated during HSC *in vitro* activation, thus suggesting that the transcriptional activity of this nuclear receptor diminishes when HSCs achieve a fully activated myofibroblast phenotype (Fig. 1B‐E), in line with the data published by Beaven et al.^21^ It is important to consider that both LXR isotypes show a different distribution in the human body, thus suggesting a potential distinct tissue‐specific or even cell‐specific role according to their localization. Moreover, they regulate an overlapping set of genes and therefore might act synergistically or compensate for each other.^19^ Mice lacking LXRα display accumulation of CEs when challenged with a high‐cholesterol diet. This proves that LXRα controls hepatic lipid metabolism through SREBP‐1c^18^, ^35^ and promotes reverse cholesterol transport and excretion.^26^ In this regard, specific analysis of primary and overexpressing HSCs revealed that LXRα (mRNA, protein content, and intracellular amount; Fig. 1C‐E) is significantly lower in HSCs with the *PNPLA3* genetic variant, in line with the histologic evaluation in human liver biopsies (data not shown). Our first findings during HSC *in vitro* activation led us to investigate the LXR downstream targets in WT and I148M HSCs. ABCA1 (Fig. 2A), SREBP‐1c, FASN, and SCD1 (Supporting Figs. S1 and S2) were diminished in HSCs carrying the *PNPLA3* genetic variant. In comparison with WT HSCs, the evidence of lower LXR activity was supported by reduced expression levels of oxysterol‐releasing enzymes CYP27A1^36^ and CH25H^36^, ^37^ (Fig. 2B), which is consistent with the hypothesis that a lack of endogenous LXR ligands contributes to their decreased expression and transcriptional activity.

Dysregulation of hepatic triglyceride metabolism and impaired cholesterol homeostasis are widely recognized as active players in the development of NAFLD. Notably, increased intracellular FC accumulation positively correlates with the transition from simple steatosis toward NASH^38^, ^39^, ^40^, ^41^ and with exacerbated HSC‐mediated liver fibrogenesis in mice fed a high‐cholesterol diet in two different models of hepatic fibrosis.^11^, ^12^ At baseline, HSCs isolated from these mice already show increased FC accumulation, which sensitizes HSCs to TLR4 signaling, leading to down‐regulation of BAMBI and a faster response to profibrogenic actions of TGF‐β.^11^ FC derives from three main sources: increased endogenous synthesis, improved extracellular uptake, and decreased efflux. Intracellular cholesterol levels finely regulate the transcriptional activity of SREBP‐2. In the nucleus, SREBP‐2 positively induces the expression of the enzymes for cholesterol synthesis and uptake, HMGCR, 3‐hydroxy‐3‐methylglutaryl coenzyme A synthase (HMGCS), and LDLR.^39^ In line with the TC and FC accumulation, our gene and protein analysis revealed that the I148M *PNPLA3*‐carrying HSCs displayed lower SREBP‐2 transcriptional activity in both primary and overexpressing cells, as suggested by decreased LDLR, HMGCR, and HMGCS expressions (Fig. 2D,E; Supporting Figs. S1 and S2). Remarkably, according to other observations,^13^ diminished ACAT1 and NPC1 expressions in HSCs carrying I148M contribute to accumulated FC in HSCs (Supporting Fig. S1), thus playing a role in liver fibrogenesis. In addition, KO HSCs for NPC1 accumulate FC and are more sensitive to TGF‐β‐induced collagen1α1 and α‐SMA expressions.^13^

Interestingly, our transfections data pointed out that LXRE transcriptional activity was impaired in I148M‐carrying HSCs, even when an LXRα plasmid was added (Fig. 3), reflecting the lower content of ligands activating this transcription factor in these cells. In particular, the contribution of diminished PPARγ signaling reported by our group^10^ and the transcriptional control of LXR by PPARγ in macrophages^29^ encouraged us to investigate whether a similar mechanism occurred in our HSCs with the *PNPLA3* variant. Indeed, only LXR activated by T09 achieved an up‐regulation comparable to that found in WT HSCs on LXRE‐luciferase without affecting the PPRE transactivation (Fig. 3C). Remarkably, WT HSCs display increased transcriptional activity of LXRE following both T09 and ROSI treatment whereas HSCs carrying the variant only following T09 treatment, underlying a possible role of PPARγ upstream of LXR (Fig. 3D). Along with the latter data, T09 treatment significantly decreased AP‐1 transcriptional activity in WT cells and had positive effects also in I148M HSCs, indicating a positive action mediated by partially reestablished LXR signaling, without affecting PPARγ downstream targets (Fig. 3). Accordingly, stimulation with LXR synthetic agonist displayed positive effects on gene expression for ABCA1 and significantly reduced collagen1α1 and CCL5 in the HSCs of both *PNPLA3* genotypes (Fig. 4, gray bars), suggesting for the first time that restoring LXR signaling in *PNPLA3* I148M variant cells could be a potential strategy to repress fibrogenic features in these HSCs (Fig. 4, right panels, black bars).

Because T09 has LXR‐independent effects mediated by FXR and PXR,^32^, ^33^ the use of LXR‐specific inverse agonist and antagonist was necessary to demonstrate that LXR was the key transcription factor involved in our findings. Indeed, LXR inverse agonist lowered ABCA1 and SREBP‐1c expression below the control levels (Fig. 5) in WT HSCs. However, in I148M HSCs, the inverse agonist and the antagonist still had effects in decreasing expression of LXR targets but they were moderate compared to WT *PNPLA3* HSCs (Fig. 5), which could possibly be caused by reduced endogenous oxysterols activating LXR.

Considering the capacity of LXRs to repress release of inflammatory mediators in macrophages, the role of these nuclear receptors strongly leads to a link between hepatic metabolism and immune response. Liver resident macrophages lacking LXRs underline that the latter control the differentiation of the Kupffer cell population and regulate immune and inflammatory responses in mice.^22^, ^41^ The activation and overexpression of LXRα following ROSI stimulation provides a direct anti‐inflammatory effect on cytokine expression in HSCs carrying the WT along with no detectable improvement in cells with the *PNPLA3* variant (Fig. 5B).

Collectively, our findings uncover I148M *PNPLA3* as an additional interesting contributor of impaired cholesterol and lipid metabolism in human HSCs, predisposing to exacerbated proinflammatory phenotype and enhanced hepatic fibrosis severity in humans. Of note, LXRα and LXRβ regulate distinct and overlapping sets of genes and show high homology in their ligand‐binding domains, thus reducing the possibilities to obtain subtype‐specific agonists. New molecules were synthetized and revealed the impact of single isotype activation in therapeutic scenarios.^27^ Novel approaches also include the use of inverse agonists for LXRs. In this regard, the SR9238 compound is effective in reducing hepatic steatosis, inflammation, and fibrosis in a model of murine steatohepatitis,^42^ highlighting the potential implications in human NASH. To counteract intracellular cholesterol accumulation, another strategy might be the use of statins. Clinical studies on patients with NAFLD have demonstrated that statins have a positive impact by diminishing steatosis, NASH, and fibrosis, although the presence of the *PNPLA3* genetic variant blunted these beneficial effects.^43^

Nevertheless, further molecular and *in vivo* studies are needed to address the specific contribution of the two LXR isotypes in HSCs expressing the *PNPLA3* variant, considering their pivotal role in controlling hepatic lipid machinery and innate immune response, and therefore leading to an alternative therapeutic approach beyond PPARγ agonists.
