# Supplementary figures and images for: PNPLA3 I148M Variant Impairs Liver X Receptor Signaling and Cholesterol Homeostasis in Human Hepatic Stellate Cells
Source: Hepatol Commun. 2019 Jul 15;3(9):1191–204. doi: 10.1002/hep4.1395 (PMC6719741; doi:10.1002/hep4.1395)

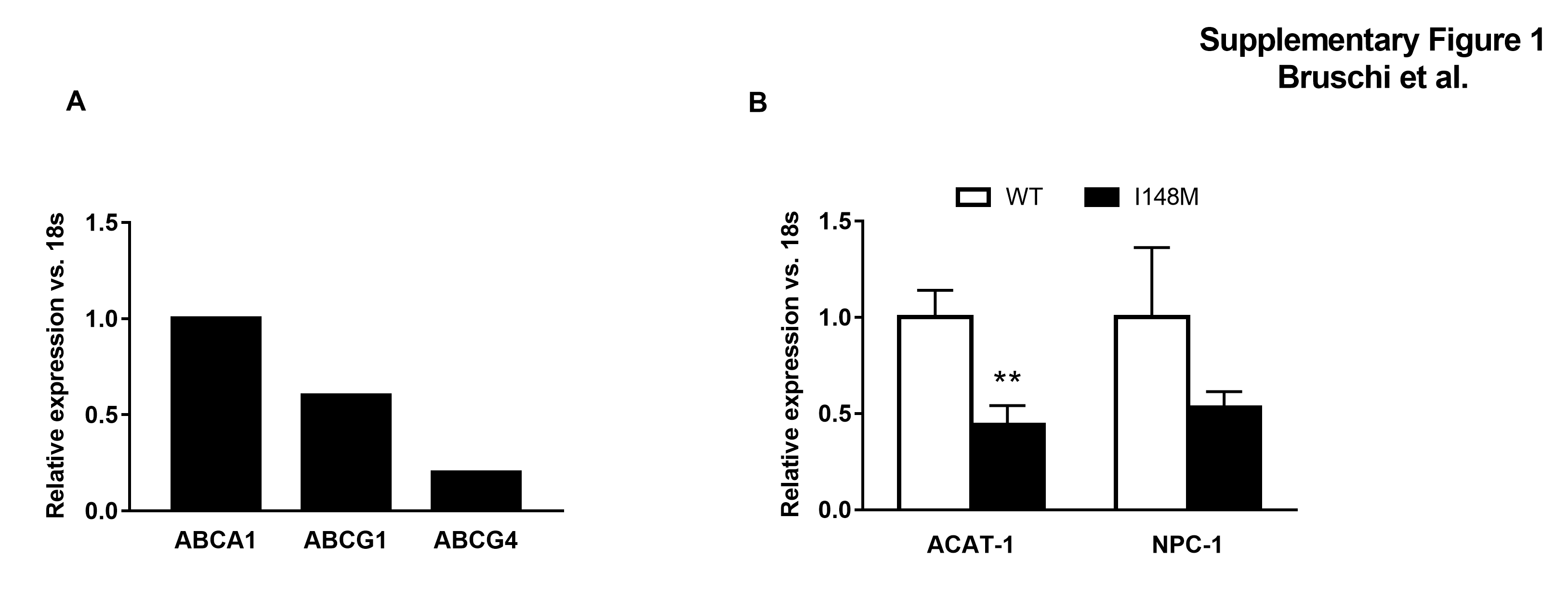

Supplement: Supplementary file 1 [file HEP4-3-1191-s001.tif]

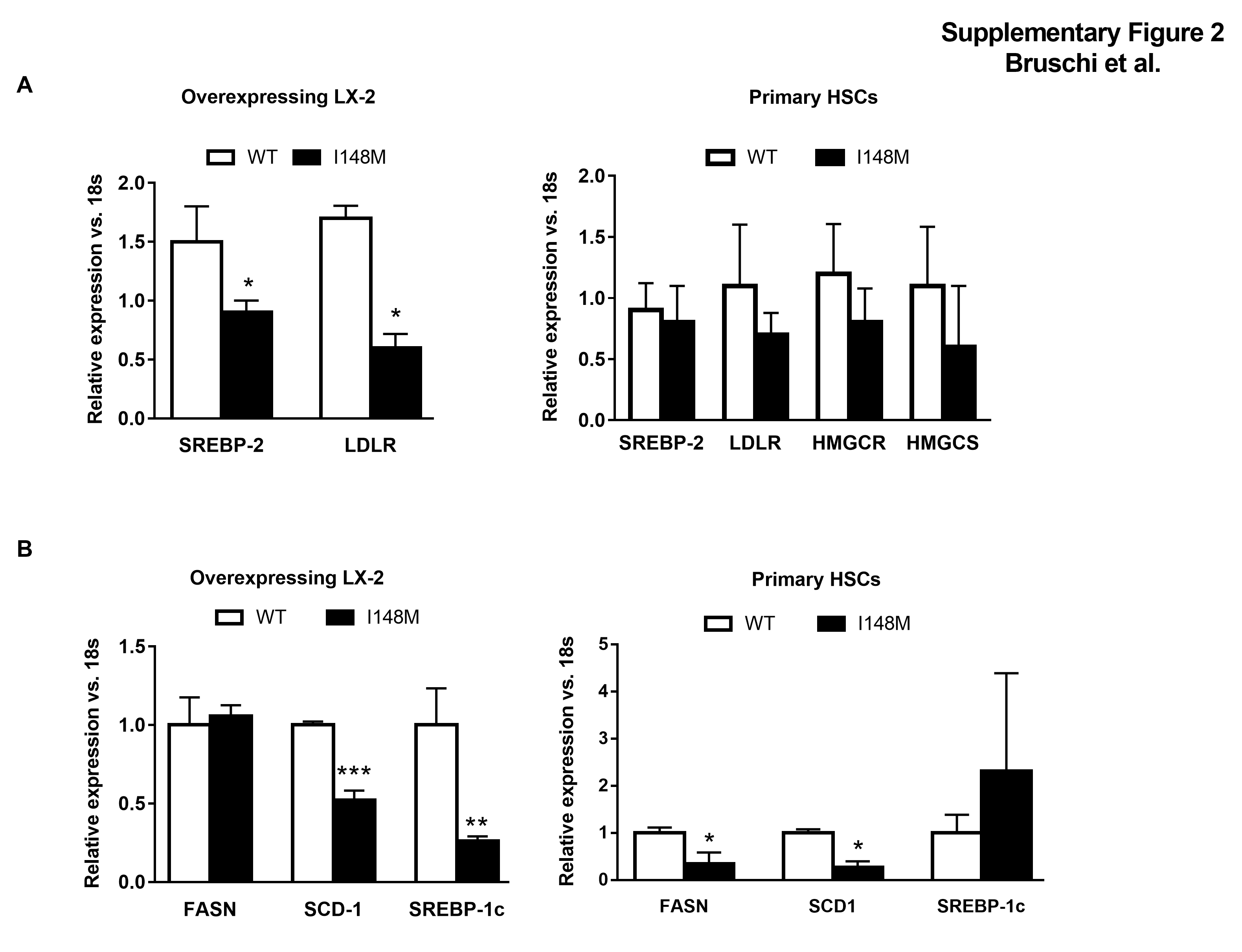

Supplement: Supplementary file 2 [file HEP4-3-1191-s002.tif]
