# Supplementary material for: PNPLA3 I148M Variant Impairs Liver X Receptor Signaling and Cholesterol Homeostasis in Human Hepatic Stellate Cells
Source: Hepatol Commun. 2019 Jul 15;3(9):1191–204. doi: 10.1002/hep4.1395 (PMC6719741; doi:10.1002/hep4.1395)
Supplement: Supplementary file 3 [file HEP4-3-1191-s003.docx]

**The PNPLA3 I148M Variant Impairs LXR Signaling and Cholesterol Homeostasis in Human Hepatic Stellate Cells**

Francesca Virginia Bruschi,^1^ Thierry Claudel,^1^ Matteo Tardelli,^1^ Patrick Starlinger,^2^ Fabio Marra,^3^ and Michael Trauner^1^

From the ^1^Hans Popper Laboratory of Molecular Hepatology, Division of Gastroenterology and Hepatology, Department of Internal Medicine III, Medical University of Vienna, Vienna, Austria; ^2^Department of Surgery, Medical University of Vienna, Vienna, Austria; ^3^Clinical Pathophysiology Department, University of Florence, Florence, Italy.

**Table of Contents:**

- Supporting Materials and Methods
- Supporting Figure Legends

**Supporting Material and Methods:**

*Immunofluorescence staining on cells:*

WT and I148M HSCs were seeded at the density of 500 cells per square millimeter on glass coverslips for 2 days. Thereafter, cells were washed in cold PBS and fixed/permeabilized in formalin 10% for 15 minutes. After blockage of not specific sites with PBS 1% goat serum and 0.05% TWEEN 20 (Sigma-Aldrich) anti-LXRα (1:200, rabbit; Novus NB300-612) and anti-LXRβ (1:200, mouse; Santa Cruz Biotechnology H-8) antibody were added in solution with PBS 1% goat serum for 1 hour. Slides were washed three times with PBS 1X Goat Alexa 488 anti-rabbit IgG and Goat Alexa 568 anti-mouse IgG (Thermo Scientific) were added together at a 1:500 dilution in PBS for 1 hour. Finally, coverslips were washed three times in PBS 1x and mounted with fluorescent mounting medium containing DAPI (Dako, USA). Immunofluorescence (IF) pictures were performed using epifluorescence microscopy (Olympus BX51 microscope).

**Supporting Figure Legends:**

**Supporting FIG. S1.** Expression of cholesterol transporters ABCA1, ABCG1 and ABCG4 differs in primary HSCs. (A,B) LX-2 stably overexpressing cells and primary human HSCs were isolated, genotyped, and cultured in vitro, as described in Materials and Methods (n = 3 for each PNPLA3 genotype). Cells were harvested, and mRNA expression of acetyl-CoA acetyltransferase (ACAT1) and Niemann-Pick disease, type C1 (NPC1) was measured by real-time PCR and normalized to 18s. Open bars show results for PNPLA3 WT HSCs and closed bars for I148M HSCs. Data are expressed as mean value ± SD. ***P* < 0.01 versus WT carriers.

**Supporting FIG. S2.** Endogenous cholesterol synthesis and uptake is decreased in HSCs with the PNPLA3 variant. (A) LX-2 stably overexpressing cells and primary human HSCs were isolated, genotyped for PNPLA3, and cultured in vitro, as described in Materials and Methods (n = 3 for each PNPLA3 genotype) were harvested, and mRNA expression of SREBP-2, LDLR, HMGCR, and HMGCS was measured by real-time PCR and normalized to 18s. (B) Cells were harvested, and mRNA expression of FASN, SCD1, and SREBP-1c was measured by real-time PCR and normalized to 18s (n = 3 for each PNPLA3 genotype). Open bars show results for PNPLA3 WT HSCs and closed bars for I148M HSCs (n = 3 for each genotype). Data are expressed as mean value ± SD. **P* < 0.05, ***P* < 0.01, and ****P* < 0.001 versus WT carriers.
